# Low Even Configurations in the First Spectrum of Ruthenium (Ru I), part 2

**DOI:** 10.6028/jres.063A.019

**Published:** 1959-12-01

**Authors:** R. E. Trees

## Abstract

A published calculation for the 4*d*^8^, 4*d* ^7^5*s*, and 4*d*^6^ 5*s*^2^ configurations of Ru I (R. E. Trees, J. Opt. Soc. Am. **49**, 838 (1959).) is repeated in steps. Displacements produced by configuration interaction are evaluated, and departures of term positions from familiar expectations in the absence of configuration interaction are explained. The weaker perturbations produced by second-order effects of the spin-orbit interaction are then determined. It is shown that the neglect of these effects in published hand computations has obscured the remarkably good agreement between theory and observation that is obtainable in spectra of the second long period. The eigenvectors are based on “third-order eigenfunctions” which describe the levels simply, and show the degree of *LS*-coupling in a more quantitative manner.

## 1. Introduction

This paper is the fourth in a series of calculations carried out for complex spectra by utilizing a digital computer [1, 2, 3.]^1^ In previous calculations the Standards Eastern Automatic Computer (SEAC) was used, but this machine is no longer available. The mathematical operations that were carried out on the SEAC are described in reference [1]. Similar codes have now been completed for the IBM 704, and they have been used to obtain the results in this paper. The code for the 704 is able to manipulate the high-order matrices that arise in these calculations in a general manner, but in particular carries out the operations described in sections 2, 3, 5, and 6 of reference [1]. An output of linear formulas that approximate any specified eigenvalues of the matrices is obtained and used directly as an input to an orthonormalization code, similar to that described in section 4 of reference [1], to obtain improved values of radial parameters.^2^

The present paper is a continuation of a previous calculation for the 4*d*^8^, 4*d*^7^ 5*s*, and 4*d*^6^ 5*s*^2^ configurations of Ru I [3], and part of the previous work has been repeated. An extension of the discussion of the agreement between calculated and observed energy levels and a simplification of the procedure for assigning designations to levels are also given.

All of the calculations have been carried out to assist with the experimental analysis of observed data [4]. The use of the calculations in the analysis of Ru I is discussed in the paper by Kessler which gives details of the experimental side of the work that has been done on this spectrum [5]. He found that even though the analysis was carried out by using a digital computer, levels were overlooked but later found after they were predicted by theory. These were levels determined from a relatively few, mostly weak combinations, and in some cases they would have had to be rejected as unreal in the absence of theoretical confirmation. This confirmation would naturally be improved if the low intensities of the combinations could also be explained by extending the calculations to include a consideration of the much more complicated odd configurations in this spectrum.

## 2. General Procedure

Radial parameters have already been obtained for Ru I [3], and these are given in table 1. By using these parameters, the calculation is repeated in four steps, (a) with configuration interaction and spinorbit interaction neglected, (b) with the configuration interaction included but spin-orbit interaction still omitted, (c) with configuration interaction and the first-order effects of spin-orbit interaction, and (d) finally with all interactions included. Step (d) is essentially the result already published, [3], but the eigenvectors in the present paper are referred to the more appropriate basis obtained in step (b). The first three steps were carried out for the even three-electron configurations of Ti II and Zr II by Ufford [6] and the first two steps describe the calculations carried out by Trees for the even six-electron configurations of Mo I [7]. These were hand computations, and this was a simplifying procedure used to determine the parameters successively in order of their decreasing effects on the positions of the levels. The rigorous approach is, of course, to include all interactions at the start and determine the parameters simultaneously, but this is a difficult calculation even with the help of digital computers. However, after the rigorous determination of parameters has been carried out [3], it is instructive to repeat the calculation by steps. This procedure will also clarify the interpretation of third long period spectra [1, 2].

A convenient terminology has been used by Ufford to describe this calculation (footnote 5 of ref. [6]). His “first-order eigenfunctions” are those in which the matrix of electrostatic interaction is diagonal in each configuration with respect to different kinds of terms occurring in LS coupling. There are pairs of duplicated terms having the same LS-values, in both *d*^7^*s* and *d*^6^*s*^2^, and the electrostatic interaction is not diagonal within the configuration for these pairs when the base vectors are distinguished by different values of Racah’s seniority number (seniority numbers are given as prefixes in table 3) [8]. The “second order eigenfunctions” are the basis in which the electrostatic interaction is also diagonal for each of these pairs (^+^ and ^−^ sign superscript postscripts are usually used to distinguish the new base vectors, but since our tables refer only to the “minus” − component, the sign has been omitted). These correspond to the classification in A.E.L., and are obtained in step (a). “Third order eigenfunctions” are ones for which the matrix of electrostatic interaction is diagonal for all three configurations *d*^8^, *d*^7^*s*, and *d*^6^*s*^2^ taken together and they are obtained in step (b). There are terms where the concept of second-order eigenfunction is not needed, but this concept is applied in general to mean second or next lower applicable order eigenfunction. Similarly, there are terms that occur only once in all three configurations for which neither the concept of third-order or second-order eigenfunction is needed (i.e., the ^5^F, ^5^P, ^1^H, and ^1^P terms from *d*^7^*s* and the ^5^D and ^1^I terms from *d*^6^*s*^2^), but the terminology will still be applied in general with a similar interpretation.

## 3. Configuration Interaction

The matrices were first generated with the spinorbit and configuration interactions omitted, by using the parameters of table 1 with zero values for the three spin-orbit parameters, zeta, and the three configuration interaction integrals (the two equal values of H_2_ and one of the two equal values of G_2_). The eigenvalues of these matrices were calculated and they are given as the “unperturbed positions” in column (1) of table 2 (terms occurring only once in all three configurations are omitted for brevity). These are the term positions that would be expected in Ru I if simple comparison were made with a spectrum having weak configuration interaction, such as Fe I. The eigenvectors are the second-order eigenfunctions; since they can be easily calculated by hand, they have been omitted for brevity.

The full matrices of electrostatic interaction were then generated, the three spin-orbit parameters being the only ones assigned zero values. The eigenvalues of the matrices obtained in this approximation are given as the “perturbed positions” in column (2) of table 2. The corresponding eigenvectors are third-order eigenfunctions and these are given in table 3.

The differences between columns (1) and (2) of table 2 are given in column (3) and represent the displacements produced by configuration interaction. In half the terms these displacements are greater than 2,000 wave numbers, and in three the displacements exceed 4,000 wave numbers, i.e., *a* ^3^P, *a* ^1^D, and *b* ^1^D. The simple procedure of comparing Ru I and Fe I demonstrates the strong interaction in *a* ^3^P very easily. However, this procedure may not show that the singlet term *a* ^1^D appears below the corresponding triplet, the *a* ^3^D, from the same parent in *d*^7^. In A.E.L. and the paper by Kessler the level at 15054 is assigned to the *a* ^3^D term and the level at 17046 is given the designation *a* ^1^D_2_; except for this, the assignments of the present paper agree with those in A.E.L. and in Kessler’s paper. The interesting perturbation of *a* ^1^D is obscured when this is done. It also leaves unexplained the appearance of 4*d*^8^ *b* ^1^D above the term 4*d*^8^ *b*^1^G, the respective positions of these being 23453 and 23393. When the data is interpreted so that *a* ^1^D is lower than *a* ^3^D, the unexpectedly high position of *b* ^1^D is explained as a compensation for the low position of *a* ^1^D.

The eigenvectors given in reference [3] were available when the classification used in A.E.L. was made, and they indicated that the assignments should be as given in this paper. However, the calculated eigenvector for the level at 15054 has an undetermined error which corresponds to a difference of 0.01 between observed and calculated *g*-values. Since the accuracy of the calculated eigenvector was in question, it was difficult to be sure of the correct designations for the levels because slight changes in the parameters sometimes produce large changes in the eigenvectors. It was not likely that the error could be large enough to call for the designations used in A.E.L., but this opinion is confirmed only now after it is established that the *a* ^1^D is perturbed by strong configuration interaction rather than by an unexpectedly large second-order spin-orbit effect. On the same theme, it may be added that it would be more consistent in both A.E.L. and this paper if the configuration assignments for *c* ^3^F and *d* ^3^F were interchanged. This would put the *a* ^1^F term based on the 4*d*^7 2^F parent above *c* ^3^F (which would then be) based on the same parent, in agreement with expectation. The third-order eigenvectors (table 3) show that by a small margin of 2 percent the overall purity would be increased if this interchange were made; i.e., *c* ^3^F has 2 percent more of *d*^7^(^2^F)*s* ^3^F in its composition than it has *d*^6^*s*^2^ ^3^F, and the reverse is true for *d* ^3^F.

## 4. Spin-Orbit Interaction

The matrices were next set up with the electrostatic and spin-orbit interactions fully included by utilizing all the parameters of table 1. The same matrices were used to obtain the results in reference [3]. Since they are referred to the first-order eigenfunctions as basis, each matrix has large off-diagonal elements arising from the configuration interaction, the largest elements being the order of 5,000 or 6,000 wave numbers. By transforming the matrices to the third-order eigenfunctions of table 3 as basis, these large elements are eliminated and only the smaller nondiagonal matrix elements arising from the spinorbit interaction remain. The largest of these elements are about 2,000 wave numbers. The diagonal elements of the transformed matrices given in column (7) of table 4, are, therefore, good approximations to the eigenvalues. The eigenvalues of the matrices are given in column (5) of table 4; since eigenvalues are, of course, independent of the vector basis, they are the same as those given in reference [3]. The eigenvectors are omitted for brevity, except that in column (10) we give the percentage composition corresponding to the dominant component, the latter being specified by column (2) of table 4, along with the eigenvectors in table 3. Tables 2 to 8 of reference [3] give the eigenvectors referred to second-order eigenfunctions as basis, and for comparison the dominant percentage composition referred to this basis is given in column (9) of table 4—in this case the component is fully specified by columns (1) and (2).

The differences between the eigenvalues and the diagonal elements of the matrices are given in column (8) of table 4; these differences are the displacements produced by second- and higher-order spin-orbit interactions. The displacements are considerably smaller than those produced by configuration interaction, but they are still too large to ignore if the best significant agreement between theory and experiment is desired. Since hand computations have ignored them, best agreement was not obtained; for instance, a mean error well over ±200 wave numbers was found for the calculation carried out in reference [7]. The differences between the observed energy levels and the calculated eigenvalues are given in column (6) of table 4. and the mean error in this case is only ±63 [3]. It is the smallest so far published for a spectrum with configurations of this complexity. This is a reflection of a fact that is still to be explained, that better agreement is obtainable for spectra of the second long period than for those of the first or third long periods, even though the theory is basically the same in all three periods.^3^

Because agreement between theory and observation is so good, further study of Ru I should lead to a better understanding of sources of error in the theory. It has already been noted that errors associated with levels all belonging to the same term are often similar in sign and magnitude, indicating that the source of error is associated with the electrostatic interactions [3]. The arrangement of table 4 brings this out more clearly. There are instances where there is a dissimilarity, but it can sometimes be explained as a result of widely differing spin-orbit displacements in the different levels of the term. For instance, the large error of 183 in calculating the position of the level at 8044 (*a* ^5^P_2_) may be associated with the fact that this level undergoes the largest displacement from second-order spin-orbit interactions (i.e., 1371 wave numbers). Unfortunately, this qualitative reasoning does not explain the large error of −582 for the level at 29979 (*b* ^3^D_3_), since the displacements are relatively small for all levels of this term. Kessler only recently located a level at 29617 which might be *b* ^3^D_1_, and the fact that the position agrees well with theory makes the discrepancy for the level at 29979 more puzzling; however, he notes in his paper that the reality of this level is poorly established and he has not included it in his tables. The large error of −1828 for the level at 24174 is provisionally ignored because the experimental data do not establish the reality beyond question.

The third-order eigenfunctions are not accurate enough for a fully quantitative understanding of the spectrum; but, as already shown, they are a simple basis that is adequate for a qualitative understanding of the term positions and energy levels. Referred to either second- or third-order eigenfunctions, the term intervals show the Landé ratio and can be characterized by a single number; in the standard notation, this is the splitting factor *ζ*(*γ*SL) [9].^4^ In column (4) of table 2 are given the values of this factor when second-order eigenfunctions are used, while in column (5) it is evaluated for the third-order eigenfunctions. These columns compare in a simple way the first-order spin-orbit splitting without and with configuration interaction. For instance, it follows from table 2 that the levels *a* ^3^P_2_ and *a* ^3^P_1_ would be separated by 610 wave numbers in the absence of configuration interaction, and by 1282 wave numbers in its presence (the observed separation is 1162 wave numbers). However, it is difficult to estimate the first order splitting from the observed data because the second-order interactions cause large departures of the intervals from the Landé ratio.

## 5. Classification

By using the third-order eigenfunctions as a basis, the purity of all levels is greater (or left unaltered for levels of terms that occur only once) as shown by comparison of columns (9) and (10) in table 4. Major increases of more than 20 percent are found in the 14 levels of three pairs of terms, namely, *a* ^3^P and *c* ^3^P; *a* ^1^D and *b* ^1^D; and *c* ^3^F and *d* ^3^F. There are only three instances where the purity is less than 50 percent whereas there were 15 instances when the second-order eigenfunctions were used as a basis. The use of the third-order eigenfunctions thus shows more clearly that the majority of the levels in this spectrum show good *LS*-coupling.

A practical advantage of this is that it simplifies the procedure of assigning designations to levels. When the eigenvector is made up of many small contributions from different states there may be instances where the calculations do not indicate a strong preference for one system of designations as compared to another. Examples of this have been discussed for the first spectrum of rhenium [2]. In Re I the best designations were chosen by implicitly referring the eigenvectors to the third-order eigenfunctions as a basis. Machine codes were not available that would do this automatically and since it was done by inspection it is not certain that the best assignments were obtained. This led to uncertainty in the assignments of 11 levels, as indicated in table IX of reference [2].

A similar, somewhat trivial example of this is found in the *c* ^3^F and *d* ^3^F terms of Ru I. As shown in column (9) of table 4, the purities of the six levels of these terms are less than 50 percent when referred to the second-order eigenfunctions. An interchange of configuration assignments for the two terms results in an average increase of 4 percent in the purity of each level (this can be shown from the data in reference [3]). But the largest average increase in purity, 7 percent, would be obtained by interchanging configuration assignments only for the two pairs of levels with *J*=2 and *J*=3 If this were done, the two ^3^F terms would not show the grouping that is expected in *LS*-coupling. This would be purely the result of using second-order eigenfunctions as a basis and this poor grouping of levels would clearly be unreasonable. When referred to the third-order eigenfunctions the purities of all levels are greater than 50 percent and the correct grouping is demonstrated automatically.

## Figures and Tables

**Table 1:** Radial parameters for Ru I*

A (*d*^8^)	14752±50
A (*d*^7^*s*)	14518±40
A (*d*^6^*s*^2^)	20874±106
B (*d*^8^)	485.7±7
B (*d*^7^*s*)	542.6±3
B (*d*^6^*s*^2^)	596.7±4
C (*d*^8^)	2412.6±60
C (*d^7^s*)	2578.6±11
C (*d*^6^*s*^2^)	2740.4±18
*ζ* (*d*^8^)	837.8±30
*ζ* (*d*^7^*s*)	915.9±16
*ζ* (*d*^6^*s*^2^)	970.6±23
G_2_	1736.0±11
H_2_	329.9±2
*α*	29.41±1

Mean error = ± 63 cm^−1^	

* R. E. Trees, table 1, reference [3].

**Table 2:** Perturbation by configuration interaction in *Ru I*

Term	Calc. term position			Landé factor × (−1)	
	(1) Unpert.	(2) Pert.	(3) Diff.	(4) Unpert.	(5) Pert.
					
(^4^F) *a* ^3^F	8468	7953	−515	382	376
*d*^8^ *b* ^3^F	11219	10289	−930	419	392
(^2^P) *a* ^3^P	15585	10681	−4904	305	641
(^2^G) *a* ^3^G	13401	13002	−399	137	138
(^4^P) *b* ^3^P	16313	14245	−2068	382	207
(^2^G) *a* ^1^G	16873	14739	−2134	……..	……..
(^2^D) *a* ^3^D	17101	16526	−575	240	223
(^2^H) *a* ^3^H	16408	16074	−334	92	92
(^2^D) *a* ^1^D	20573	13975	−6598	……..	……..
*d*^8^ *c* ^3^P	18211	21795	3584	419	337
*d*^6^*s*^2^ *c* ^3^F	24038	22315	−1723	86	17
*d*^6^*s*^2^ *b* ^3^H	22574	22909	335	97	97
*d*^8^ *b* ^1^G	22108	23030	922	……..	……..
*d*^8^ *b* ^1^D	18297	22693	4396	……..	……..
*d*^6^*s*^2^ *d* ^3^P	23627	26348	2721	1411	1320
(^2^F) *a* ^1^F	27490	25481	−2009	……..	……..
*d*^6^*s*^2^ *b* ^3^G	25263	25663	400	146	145
(^2^F) *d* ^3^F	24018	26904	2886	−76	24
*d*^6^*s*^2^ *b* ^3^D	29028	29392	364	−81	−43

Terms having a parent indicated in the designation belong to the *d*^7^*s* configuration.

**Table 3:** Eigenvectors of motrices of electrostatic interaction in *Ru* I

	4*d*^7^5*s*		4*d*^8^	4*d*^6^5*s*^2^	

	(^2^P) ^3^P	(^4^P) ^3^P	^3^P	4^3^P	2^3^P
	
*a* ^3^P	.7367	−.2166	.5576	.2210	−.2250
*b* ^3^P	.2249	.8877	.2113	−.3325	.0790
*c* ^3^P	−.6048	−.0540	.7926	−.0536	−.0166
*d* ^3^P	−.1998	.3838	−.0909	.6977	−.5637
*e* ^3^P	.0320	.1222	.0894	.5924	.7906

	(3^2^D) ^1^D	(1^2^D) ^1^D	^1^D	4^1^D	2^1^D
	
*a* ^1^D	−.6028	.2448	.7249	.1949	−.1145
*b* ^1^D	.6357	−.2366	.6851	−.2629	.0378
*c* ^1^D	.0601	−.6138	.0119	.6689	−.4148
*d* ^1^D	.4785	.6998	.0017	.5110	−.1422
*e* ^1^D	−.0001	−.1326	.0699	.4294	.8906

	(^2^F) ^3^F	(^4^F) ^3^F	^3^F	4^3^F	2^3^F
	
*a* ^3^F	−.0401	.9803	.0759	.1710	−.0483
*b* ^3^F	−.2029	−.0998	.9640	.0616	−.1258
*c* ^3^F	.6881	.1308	.2340	−.6140	.2786
*d* ^3^F	.6954	−.1026	.0552	.6411	−.3030
*e* ^3^F	−.0094	−.0363	.0849	.4230	.9014

	(^2^G) ^1^G	^1^G	4 ^1^G	2 ^1^G
	
*a* ^1^G	.8926	.4312	−.1061	−.0776
*b* ^1^G	−.4087	.8670	.2191	−.1826
*c* ^1^G	.1642	−.2354	.8047	−.5197
*d* ^1^G	.0962	.0835	.5415	.8310

	4*d*^8^	4*d*^6^5*s*^2^	

	^1^S	4 ^1^S	0 ^1^S
	
*a* ^1^S	.3482	.8306	−.4347
*b* ^1^S	.9300	−.3643	.0488
*c* ^1^S	.1178	.4212	.8993

	4*d*^7^ 5*s*		4*d*^6^ 5*s*^2^

	(3^2^D) ^3^D	(1^2^D) ^3^D	4^3^D
	
*a* ^3^D	.8912	−.4018	.2103
*b* ^3^D	−.1188	.2408	.9633
*c* ^3^D	.4377	.8835	−.1669

	(^2^F) ^1^F	4 ^1^F
	
*a* ^1^F	.9265	.3762
*b* ^1^F	−.3762	.9265

	(^2^G) ^3^G	4 ^3^G
	
*a* ^3^G	.9841	−.1776
*b* ^3^G	.1776	.9841

	(^2^H) ^3^H	4 ^3^H
	
*a* ^3^H	.9752	.2213
*b* ^3^H	−.2213	.9752

**Table 4:** Second-order spin-orbit perturbations and purity of levels in *Ru* I

(1) Config.	(2) Desig.	(3) *J*	(4) Obs.	Eigenvalue		Diag. element		Purity	
				(5)* Calc.	(6)* Diff.	(7) Calc.	(8) Diff.	(9)* One conf.	(10)† Mixed conf.
									
		5	0	−17	−17	150	−167	98.67	………..
		4	1191	1166	−25	1295	−129	98.38	………..
*d*^7^(^4^F)*s*	*a* ^5^F	3	2092	2073	−19	2211	−138	98.77	………..
		2	2713	2703	−10	2898	−195	98.56	………..
		1	3105	3102	−3	3356	−254	98.24	………..
		4	6545	6530	−15	6826	−296	79.24	84.32
*d*^7^(^4^F)*s*	*a* ^3^F	3	8084	8083	−1	8329	−246	65.77	69.97
		2	9184	9167	−17	9456	−289	73.34	74.95
		4	7483	7413	−70	7549	−136	85.24	………..
		3	8575	8502	−73	8520	−18	72.03	………..
*d*^6^*s*^2^	*a* ^5^D	2	9058	9047	−11	9248	−201	59.50	………..
		1	9073	9165	92	9733	−568	57.24	………..
		0	9492	9503	11	9976	−473	91.22	………..
		3	8771	8812	41	8911	−99	96.91	………..
*d*^7^(^4^P)*s*	*a* ^5^P	2	8044	8227	183	9598	−1371	35.73	………..
		1	9620	9619	−1	10056	−437	55.82	………..
		4	9121	9084	−37	9114	−30	91.91	97.48
*d*^8^	*b* ^3^F	3	10655	10682	27	10681	1	92.03	99.41
		2	11447	11481	34	11856	−375	77.05	82.74
		2	10624	10660	36	10040	620	18.61	45.13
*d*^7^(^2^P)*s*	*a* ^3^P	1	11786	11766	−20	11321	445	40.60	70.11
		0	11753	11733	−20	11962	−229	57.20	82.91
		5	12207	12244	37	12451	−207	89.16	91.94
*d*^7^(^2^G)*s*	*a* ^3^G	4	12817	12802	−15	13140	−338	77.63	79.82
		3	13699	13697	−2	13690	7	94.89	98.01
		2	13646	13670	24	14038	−368	48.55	62.70
*d*^7^(^4^P)*s*	*b* ^3^P	1	13982	14044	62	14452	−408	75.12	83.16
		0	14828	14904	76	14659	245	84.38	88.29
*d*^7^(^2^G)*s*	*a* ^1^G	4	14700	14691	−9	14739	−48	61.15	76.45
		3	16191	16239	48	16080	159	93.69	97.55
*d*^7^(^2^D)*s*	*a* ^3^D	2	17046§	17055	9	16750	305	81.19	85.50
		1	16713	16723	10	17196	−473	69.47	73.08
		6	15550	15609	59	15614	−5	94.89	99.96
*d*^7^(^2^H)*s*	*a* ^3^H	5	16240	16270	30	16166	104	86.65	91.90
		4	17097	17101	4	16625	−476	77.66	83.25
*d*^7^(^2^D)*s*	*a* ^1^D	2	15054§	14970	−84	13975	995	21.46	52.03
*d*^7^(^2^H)*s*	*a* ^1^H	5	20056	20080	24	19880	200	94.55	………..
*d*^7^(^2^P)*s*	*a* ^1^P	1	20242	20224	−18	19057	1167	73.27	………..
		2	20934	20934	0	21458	−524	40.50	66.43
*d*^8^	*c* ^3^P	1	22293	22282	−11	22133	149	61.13	97.84
		0	24174?	22346	−1828	22470	−124	62.41	98.09
		4	21643	21703	60	22264	−561	37.31	57.89
*d*^6^*s*^2^	*c* ^3^F	3	22419	22246	−173	22332	−86	43.65	96.46
		2	22343	22194	−149	22383	−189	38.16	95.31
		6	22162	22243	81	22425	−182	92.50	97.58
*d*^6^*s*^2^	*b* ^3^H	5	22519	22595	76	23006	−401	78.40	84.15
		4	23005	22997	−8	23490	−493	48.56	52.55
*d*^8^	*b* ^1^G	4	23393	23397	4	23030	367	60.43	75.62
*d*^8^	*b* ^1^D	2	23453	23485	32	22693	792	31.40	64.34
		2	24927	24887	−40	25028	−141	70.29	89.01
*d*^6^*s*^2^	*d* ^3^P	1	27561	27538	−23	27669	−131	67.18	82.72
		0	………..	27948	………..	28989	−1041	62.26	77.46
*d*^7^(^2^F)*s*	*a* ^1^F	3	25201#	25210	9	25481	−271	57.72	69.02
		5	25603	25562	−41	25082	480	82.28	84.75
*d*^6^*s*^2^	*b* ^3^G	4	25643	25655	12	25808	−153	52.27	53.13
		3	26076	26031	−45	26389	−358	46.44	46.77
		4	27289	27311	22	26833	478	29.28	62.15
*d*^7^(^2^F)*s*	*d* ^3^F	3	27517	27535	18	26928	607	24.68	65.26
		2	26815#	26875	60	26999	−124	42.58	97.19
		1	29617?#	29621	4	29264	357	76.96	83.34
*d*^6^*s*^2^	*b* ^3^D	2	29352	29397	45	29350	47	83.43	90.12
		3	29979	29397	−582	29478	−81	91.23	98.06
*d*^6^*s*^2^	*a* ^1^I	6	29677#	29788	111	29601	187	97.55	………..

* R. E. Trees, tables 2 to 8, reference [3].

† Mixture of configurations defined by eigenvectors in table 3.

§ These two levels have designations interchanged from those given in A.E.L.

# These levels given by Kessler, reference [5].

## Footnotes

1 Figures in brackets indicate the literature references at the end of this paper.

2 The orthonormalization code has been written by P. J. Walsh and E. V. Haynsworth and is in general use at the National Bureau of Standards.

3 Racah has made calculations for many spectra of the first and second long periods and has also found that exceptionally close agreement is obtained in spectra of the second long period (private communication).

4 The term interval between two levels with consecutive *J*-values is equal to the product of this factor and the larger of the *J*-values. The level of larger *J*-value is higher if this product is positive, and lower if it is negative.
